# Supplementary material for: Degradation Behavior and Mechanical Integrity of a Mg-0.7Zn-0.6Ca (wt.%) Alloy: Effect of Grain Sizes and Crystallographic Texture
Source: Materials (Basel). 2022 Apr 26;15(9):3142. doi: 10.3390/ma15093142 (PMC9102660; doi:10.3390/ma15093142)
Supplement: Supplementary file 1 [file materials-15-03142-s001.zip › materials-1668285-supplementary.pdf]

1 Phase Diagram

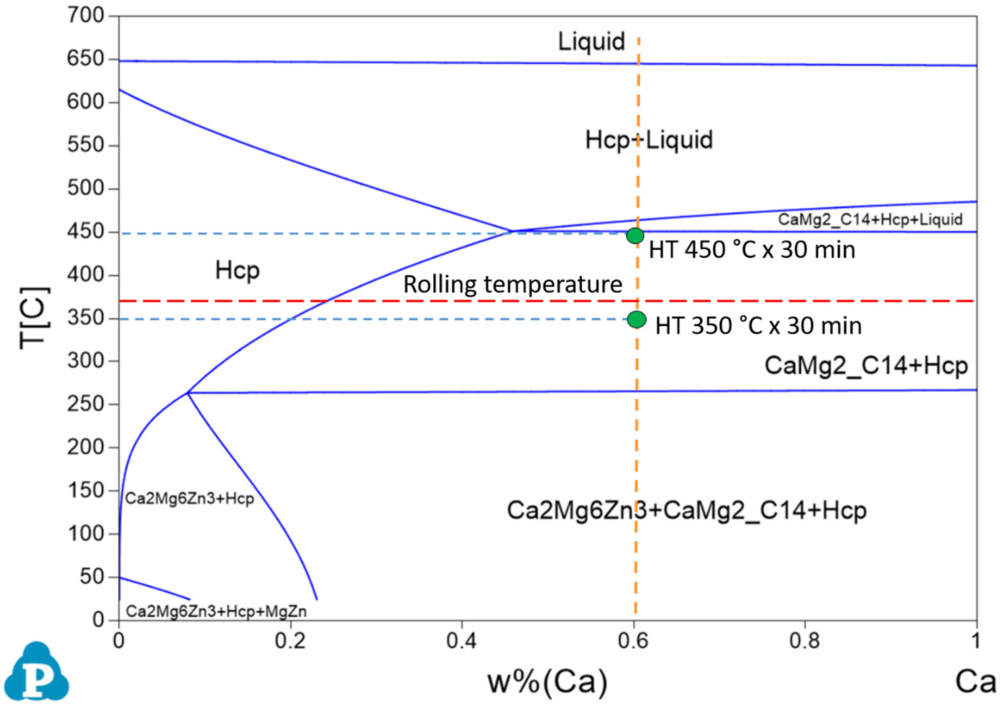

Figure S1. Pseudo-binary Mg-0.7Zn-1Ca phase diagram showing the rolling and annealing temperatures of ZX11-350 and ZX11-450 samples

2 Methodology

2.1 Hydrogen evolution test

Previously to any measurement a calibration curve was done in order to relate the amount of H_2 moles generated during the immersion with the area of the chromatographic peak. With the gas ideal equation, it is possible to relate via the calibration curve, the integrated area of the chromatographic hydrogen peak at different known pressures (using pure H_2 gas) and thus determine the moles of H_2 generated.

$$n = P\,(V_{total} - V_{solution})/RT \quad (2)$$

Where, n is the number of moles of H_2, P is the pressure of the system at each H_2 injection entering in the closed system, V_{total} is the total volume of the closed system reactor and $V_{solution}$

is the volume of the reaction solution (50 mL of DMEM/F-12 +10% v/v FBS), R is the ideal gas constant (0.082 L atm/ mol K) and T is the reaction temperature (310.15 K or 37 °C). Then, the amount of H_2 moles evolved for the ZX11-350 and -450 conditions is stablished considering that 1 mol of hydrogen gas corresponds to the dissolution of 1 mol of magnesium [1].

2.2 Cytotoxicity assay (methodology)

Mesenchymal stem cells isolation and characterization: Mesenchymal stem cells derived from human adipose tissue (MSC-Ad) were obtained according to Sanchez-Sanchez et al. [12]. Briefly, subcutaneous adipose tissue was obtained under Informed Consent from patients undergoing selective liposuction aesthetic surgeries. The Informed Consent and experimental protocols were approved by the Institutional Committee on Human Research of the Instituto Nacional de Rehabilitacióon Luis Guillermo Ibarra Ibarra (INR), Mexico (Project registry INR-90/17). Lipoaspirate samples were digested at 37 °C in DMEM/F-12 containing 0.1% type I collagenase (Worthington Biochemical). Then, cells were passed through a 70 μm strainer, centrifuged and seeded at 40,000 cells/cm2 in culture flasks with Dulbecco's Modified Eagle's Medium/Ham F-12 50/50 Mix (DMEM/F-12; Cat. 10092-CV, Corning®, USA) supplemented with 10% v/v Fetal Bovine Serum (FBS; Cat. 160000 44-PRO, Gibco®, USA) and 1% penicillin/streptomycin/amphotericin (anti-anti; Cat. 15240062, Gibco®; USA). After 24 h, floating cells were removed and the adherent MSC-Ad were cultured to confluence. Cells were maintained and subcultured (up to passage 4) in supplemented DMEM/F-12 supplemented with 10% v/v FBS and 1% v/v anti-anti, under standard cell culture conditions. Finally, cells were collected to be used in all experiments as cells in passage 2-4.

Collected MSC-Ad were characterized by their differentiation potential by culturing with conditioning media using the Stem Pro® Chondrogenesis (A1007101), Osteogenesis (A1007201) and Adipogenesis (A1007001) Differentiation kits (Gibco®), according to the

manufacturer protocols. After culturing, independent cell cultures were processed for evaluation by qualitative Alcian Blue and Safranin O staining assays for chondrogenesis differentiation, qualitative and quantitative Alizarin Red and qualitative Von Kossa staining assays for osteogenesis, and Oil Red quantitative and qualitative staining assays for adipogenesis. For verification of MSC markers, independently, in first and four passages, MSC-Ad were collected and suspended to a concentration of 0.2 × 106 cells in Phosphate Buffer Saline 1X (PBS; Gibco®) supplemented with 0.5% FBS. Aliquots with 50 μL aliquots of the cells suspension were transferred to flow cytometry tubes and incubated for 45 min at 4 °C with CD34-PE (BD-550761), CD45-FITC (BD 555482) and HLA-DR-APC (BD-559866) negative markers, and CD105-PE (BD 560839), CD73-APC (BD 560847) and CD90-FITC (BD 555595) positive markers, all acquired from BD Bioscience, BD Pharmingen. Negative control staining was performed using a FITC-conjugated mouse IgG1 isotype, PE-conjugated mouse IgG1 isotype, and APC-conjugated mouse IgG1 isotype antibody (all from BD Biosciences). Subsequently, cells were washed with PBS and diluted in 500 μl of PBS and characterized using a FACSCalibur cytometer (Becton Dickinson) equipped with a laser BLUE 488 nm. Data analysis was performed with the Cell Quest Pro software (Becton Dickinson Immunocytometry Systems).

2.3 Mesenchymal Stem Cell expression

This analysis demonstrated that the adipose obtained cells have the potential to differentiate and therefore can be considered mesenchymal stem cells.

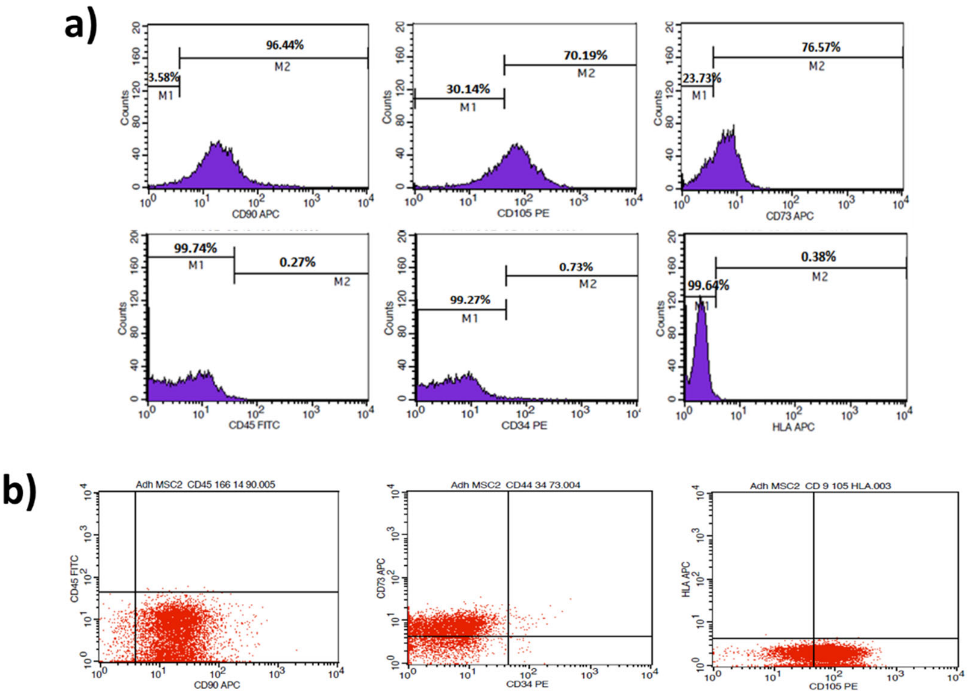

Figure S2. Adipose tissue derived mesenchymal stem cells in passage 2. A) Positive expression for mesenchymal stem cells surface markers CD90, CD105 and CD73, and lack of expression for hematopoietic markers CD45, CD34 and HLA. B) Percentage of simultaneous positive/negative expression for CD45-CD90, CD73-CD34 and HLA-CD105.

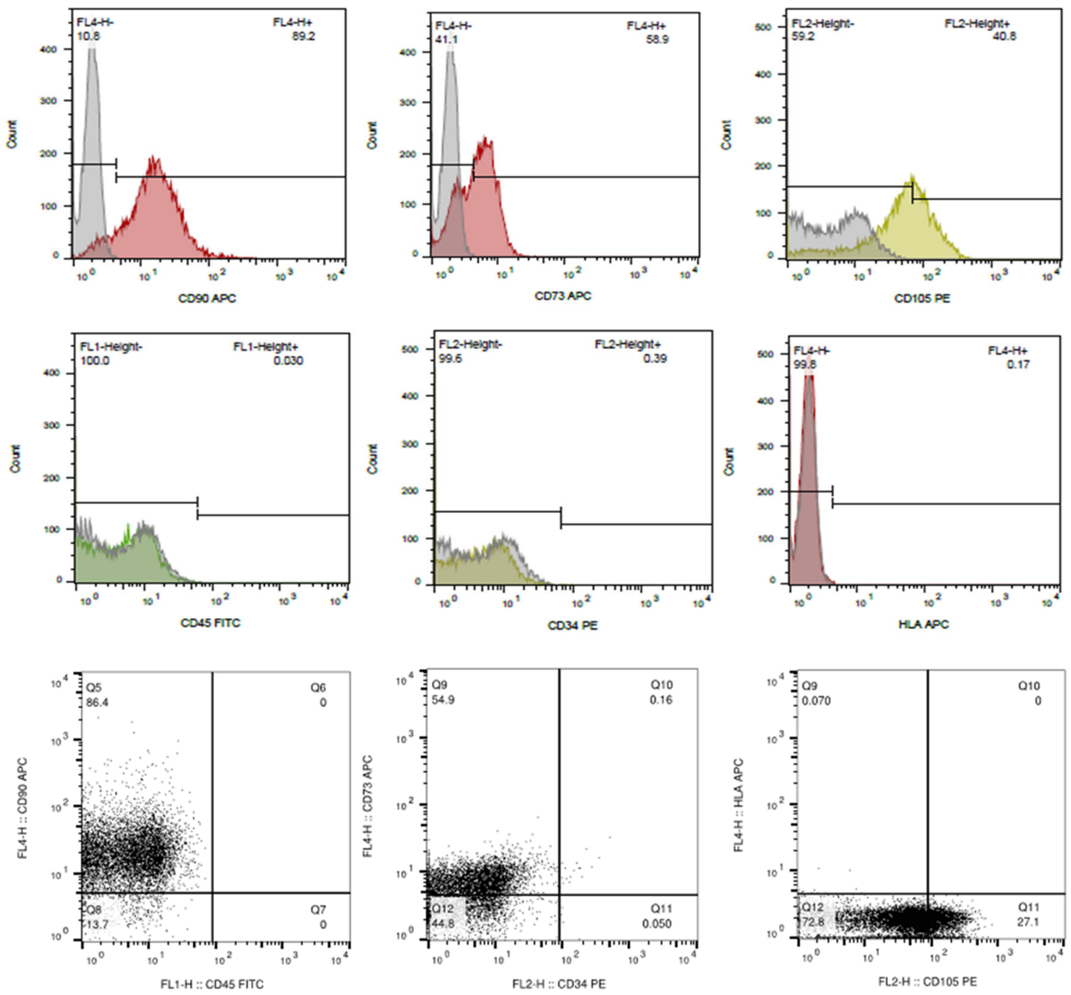

Figure S3**.** Adipose-derived mesenchymal stem cells in passage 4. A) Positive expression for mesenchymal stem cells surface markers CD90, CD73 and CD105, and lack of expression for hematopoietic stem cells markers CD45, CD34 and HLA. B) . B) Percentage of simultaneous positive/negative expression for CD90-CD45, CD73-CD34 and CD105-HLA.

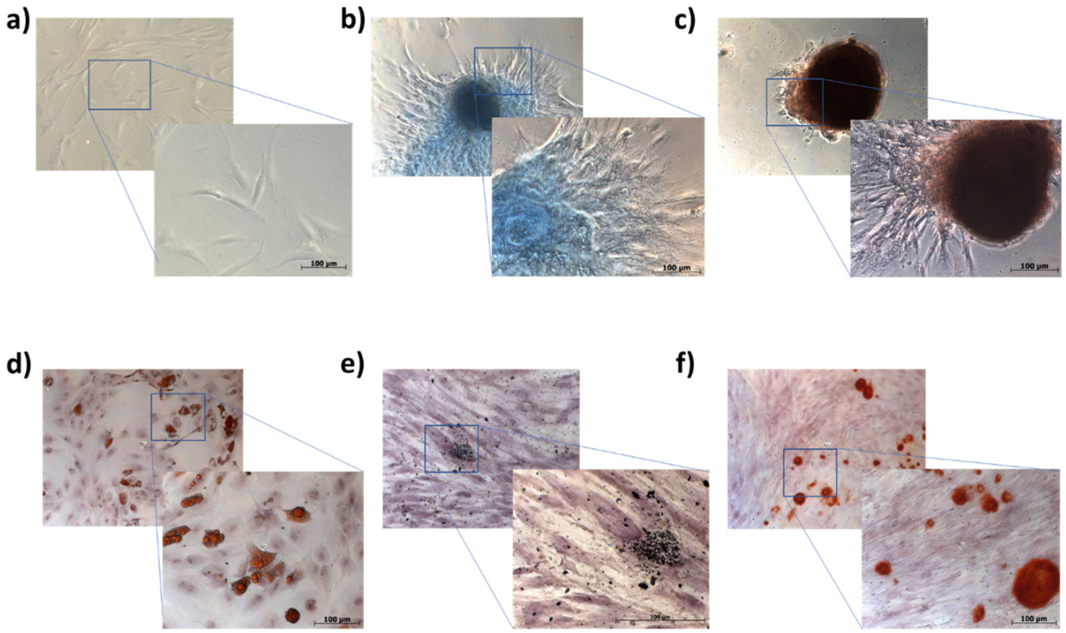

Figure S4. Characterization of Adipose-derived Mesenchymal Stem Cells in pass 4, after culture in conditioning differentiation media. A) Morphology of adhered cells, and B) Alcian Blue, C) Safranine O, D) Oil red, E) Von Kossa and F) Alizarin red staining, after culture in chondrogenesis, adipogenesis and osteogenesis differentiation media, respectively.

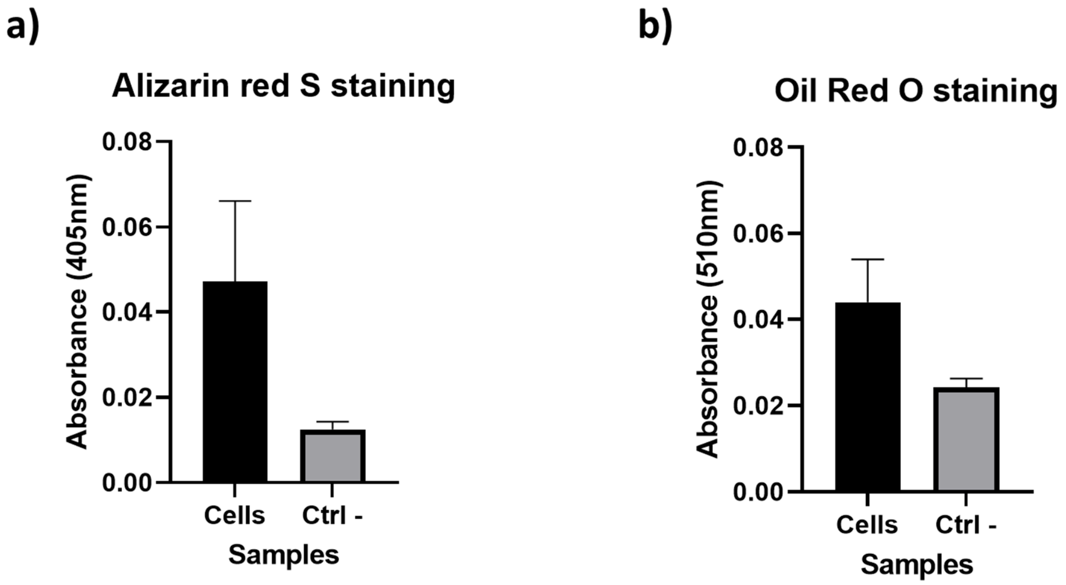

Figure S5. Quantitative characterization of differentiation potential of Adipose-derived Mesenchymal Stem Cells in pass 4. A) Alizarin red S dye, B) Oil Red O dye colorimetric detection, extracted from de stained cell monolayer after culturing in osteogenesis and adipogenesis differentiation media, respectively. Ctrl- corresponds to cells incubated with no-conditioning fresh supplemented DMEM/F12.

3 Results

The first arc, at high frequencies, is associated with the resistance of the corrosion products formed on the alloy surface, the middle arc corresponds to the double layer and charge-transfer resistance of the samples, and the arc at low frequencies is related to adsorption/desorption of intermediates species and pitting corrosion [29]–[31]. The quantitative analysis of the EIS data using an equivalent circuit indicates a total resistance of $3.87x10^3$ Ωcm^2 and 4.01x103 Ωcm^2. The equivalent circuit considered to fit the EIS experimental data is shown in Fig. S7, where Rs is the solution resistance, Rf is the resistance of the corrosion products layer, and CPE_f is the constant phase element related to the capacitance of the passive layer formed on the sample's surface. The R_t and CPE_t represent the charge transfer resistance and double-layer capacitance. Finally, an inductance element L and its respective resistance R_L are included to fit the response at low frequencies, commonly attributed to the corrosion nucleation at the initial stage of localized corrosion (pitting corrosion) and

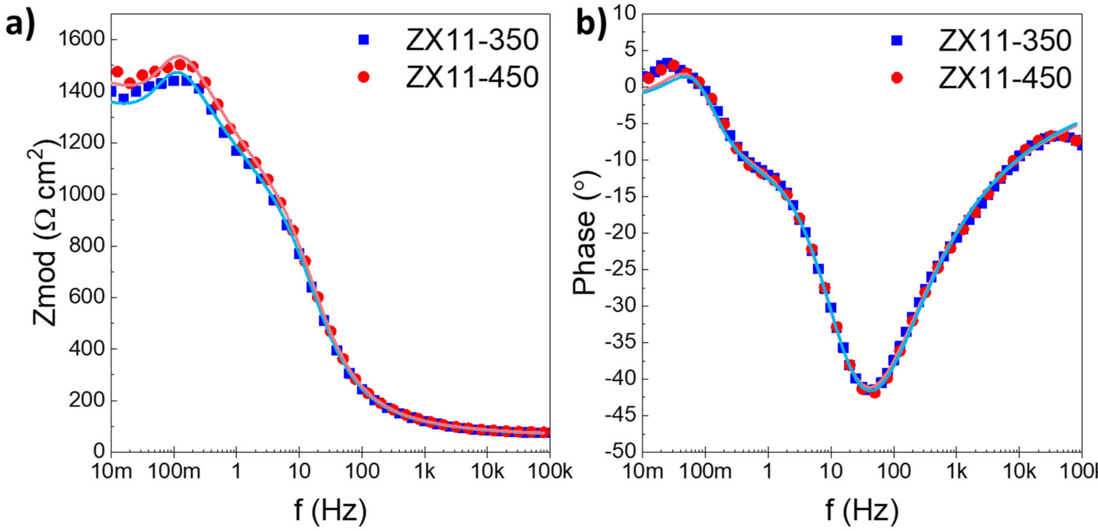

adsorption/desorption of intermediate species on the alloy surface [32], [33]. The fitted parameters are shown in Table S1.

Figure S6. Bode plots of ZX11 alloy using DMEM/F-12 supplemented with 10% v/v FBS as electrolyte. For the EIS data, the symbols correspond to the experimental data and the lines to the fitting. All the experiments were done by triplicate at least, observing a good reproducibility.

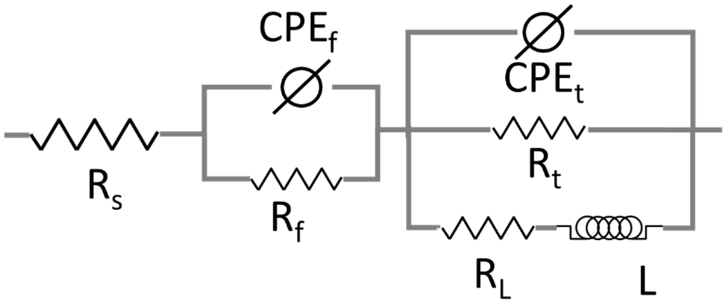

Figure S7. Equivalent circuit considered to fit the EIS experimental data of ZX11 alloy using DMEM/F-12 +10% v/v FBS as electrolyte.

Table S1. Fitted parameters of the equivalent circuit for ZX11-350 AND ZX11-450 samples. Total resistance is calculated adding all the resistances.

	**ZX11-350**	**error**	**ZX11-450**	**error**
R_s (Ω cm^2)	61.62	1.81	64.45	1.80
CPE_f (S s^a/cm^2)	$3.17x10^{-5}$	$1.2x10^{-6}$	$2.9x10^{-5}$	$3.0x10^{-6}$
a1	$8.91x10^{-1}$	--	$9.0x10^{-1}$	---
R_f (Ω cm^2)	678.8	26.11	680.8	51.4
CPE_{dl} (S s^a/cm^2)	$8.8x10^{-4}$	$1.09x10^{-4}$	$7.7x10^{-4}$	$1.2x10^{-4}$
a2	$3.4x10^{-1}$	$1.7 x10^{-2}$	$3.6x10^{-1}$	$1.8x10^{-2}$
R_t (Ω cm^2)	$1.93x10^3$	$3.0 x10^2$	$1.98x10^3$	$2.7x10^2$
R_L (Ω cm^2)	$1.21 x10^3$	$1.3 x10^2$	$1.40x10^3$	$1.9x10^2$
L (H cm^2)	$3.5 x10^3$	--	$3.5 x10^3$	---
Goodness of Fit	$4.17 x10^4$		$4.7x10^4$	
Total resistance (Ω cm^2)	$3.87 x 10^3$		$4.01 x10^3$	

3.1 Hydrogen evolution

Here we compare the amount of evolved hydrogen when the experiment is done with and without fetal bovine serum, which is a protein-rich component. The results of degradation rate measured from the hydrogen evolution test in terms of hydrogen moles produced and mass loss of Mg per unit of area are displayed in Figure S4a for ZX11-350 and ZX11-450 samples during 4 h in DMEM/F-12 and DMEM/F-12 with 10% v/v of FBS. In the case of DMEM/F-12 the hydrogen evolution increased, obtaining a hydrogen evolution rate of 4.40 and 4.75 μmol/ cm^2 h during 4h, equivalent with the mass loss of Mg rate of 0.10 and 0.11 mg/cm^2 h (see Table S2) for ZX11-350 and ZX11-450 respectively, with a relatively small variation between samples. To determine the influence of proteins on hydrogen evolution and mass loss of both conditions, DMEM/F-12 was supplemented with 10% v/v FBS. With the addition of FBS, a considerable increase in the amount of hydrogen generated can be observed, obtaining a hydrogen evolution rate of 8.31 and 7.81 μmol/ cm^2 h during 4h, equivalent with the mass loss of Mg rate of 0.202 and 0.189 mg/cm^2 h for ZX11-350 and ZX11-450 respectively (see Table S2).

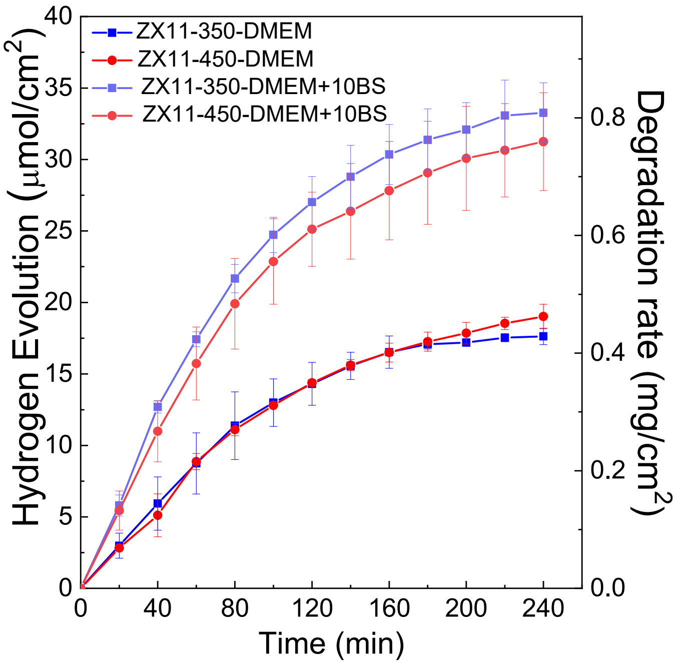

Figure S8. Hydrogen evolution measurements of the ZX11-350 and -450 in DMEM/F-12 with and without FBS.

Table S2. Average and Instantaneous corrosion rates estimated from the HE measured by gas chromatography in a closed system consisting of a 127 mL glass reactor with 50 mL with a solution of DMEM/F-12 (P_{AH}*) or DMEM/F-12 + 10% v/v FBS (P_{AH}) at 37 °C during 4 h of immersion.

	**P_{AH}***	**P_{AH}**	**P_{AH}***	**P_{AH}**	**P_{AH}***	**P_{AH}**	**P_{AH}***	**P_{AH}**
	(μmol/ cm^2 h)		(mg/cm^2 h)		(ml/cm^2 h)		(mm/year)	
**ZX11-350**	4.40	8.31	0.107	0.202	0.11	0.21	0.25	0.48
**ZX11-450**	4.75	7.81	0.115	0.189	0.12	0.20	0.27	0.45

To explain this effect, many authors have observed that the addition of proteins in DMEM delays the formation of a protective layer from corrosion products [2]–[4]. In particular, the synergistic effect between adsorption, the addition of protein in pH, and the chelating effect directly modifies magnesium alloys' corrosion [4]. Furthermore, it has been observed that proteins increase osmolality, releasing more Mg^{2+} ions from the sample. Therefore, under cell culture conditions, the addition of proteins increases the corrosion rate [5], which indicates a significant contribution of the proteins in the corrosive response of the system. The protein's

role in the corrosion response of metals depends on the alloy, proteins type, and immersion time [6]. Hohn et al. [7] present a review about the adsorption of proteins on Mg alloys.

Furthermore, Johnson et al. reported that the chelation process on the metallic ions and oxides in proteins-containing solutions could accelerate the degradation rate of the Mg-Y alloy [8]. Thus, we conclude that as proteins bond with the Mg^{2+} ions resulting from the corrosion process, the passivating corrosion products film is not formed on the sample's surface. Consequently, the degradation rate is higher in the presence of proteins in the solution. It is important to note that 10% v/v FBS is added to the culture medium at 37 ° C to simulate conditions similar to blood plasma.

3.2 Degradation products characterization

The products from the degradation of the ZX11 samples in the medium were observed by SEM, and their composition and structure were determined by EDS and XRD. Figure S5(a-b) shows the morphology of the ZX11-350 sample's surface with degradation products resulting from the immersion test at 6 days of immersion. The images were taken at different magnifications. Although only one sample is shown, both ZX11-350 and ZX11-450 had the same appearance. In the SEM images, four kinds of morphologies are distinguished. These are distributed in different zones denoted as A, B, C, and D. EDS evaluation was done in each zone, the results are included in Fig. S5, and the elemental composition provides insights into the compounds' nature. Zone A presents petal-like precipitates, a similar morphology was reported before, but the composition was not revealed [9]. Zone B shows minor corrosion precipitates spread on the alloy surface with Na, P, and Ca detection by EDS. The low amount

implies that these particles might correspond to the precipitation of some salts from the DMEM/F-12. Flat, circular, and cracked regions are observed in zone C. This is the typical morphology of magnesium hydroxide reported by other authors [10]–[12]. Zone D shows another morphology, which we believe is correlated with the products in zone C. Additionally, a cilia-shaped region can be observed in the insert of zone D, and it could be related to a chlorine-based salt. The cracks in the products of zones C and D could result from the mismatch between the lattice parameters of the alloy and the compounds, which would affect the adherence and facilitate the detachment of the degradation products, hence allowing further degradation of the ZX11 alloy.

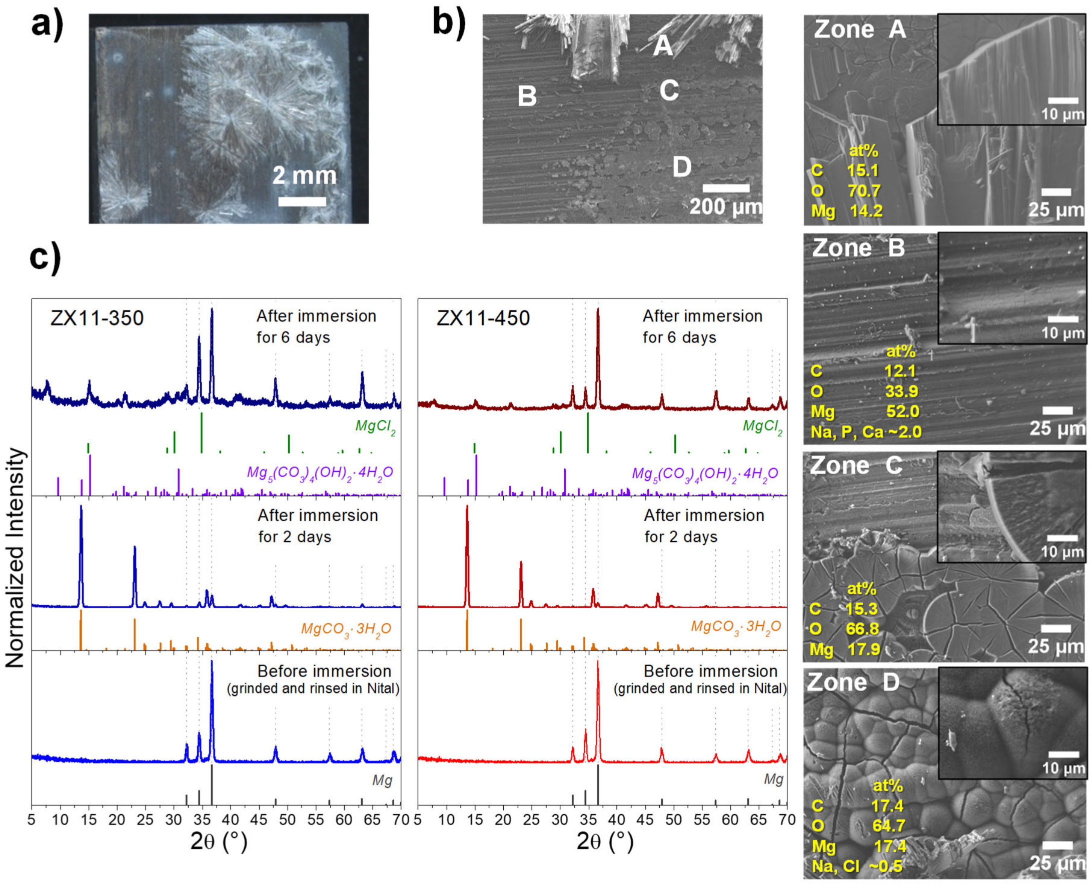

Figure S9. (a) Representative macroscopic images of ZX11-350 condition after 6 days of immersion in DMEM/F-12 supplemented with 10% v/v FBS. (b) Representative micrographs by SEM of the same sample where different surface morphologies and degradation products

are distinguished, these are denoted as zones A, B, C and D. (c) XRD patterns of ZX11-350 and ZX11-450 conditions before, and after immersion of 2 and 6 days in DMEM/F-12 supplemented with 10% FBS.

The XRD patterns of the ZX11-350 and ZX11-450 conditions before the immersion test, located at the bottom of Fig. S5(c), show only the crystalline structure of Mg matched with the JCPDS card No. 00-035-0821. The typical intermetallic phases in these alloys, Mg_2Ca and $Ca_2Mg_6Zn_3$ [13], were not detected by XRD. Regarding the patterns corresponding to the samples after immersion in DMEM/F-12 supplemented with 10% v/v FBS at different immersion times, it can be observed that the diffractograms are different, i.e., the corrosion products changed along the immersion time. No significant differences between the position of the peaks in the diffractograms of ZX11-350 and ZX11-450 were observed for each immersion time. One of the degradation products detected on the sample's surface after immersion for 2 days was identified as $MgCO_3 \cdot 3H_2O$ using the JCPDS card No. 01-070-1433. A different degradation product was identified in the samples immersed for 6 days. The main product corresponded to $Mg_5(CO_3)_4(OH)_2 \cdot 4H_2O$, which was identified using the JCPDS card No. 01-070-036. This is a more complex degradation product compared to the magnesium carbonate-based compound identified in the samples immersed for 2 days. A secondary product was identified in the diffractograms corresponding to the alloys immersed for 6 days, and this was $MgCl_2$ with JCPDS card No. 00-025-1156.

The formation of $MgCl_2$ could result from the dissolution reaction of the hydroxide with the Cl^- ions in the immersion medium. This identification was based on the detection of Cl by EDS. The peaks of Mg are more intense in the patterns of the samples immersed for 6 days than those in the samples immersed for 2 days. A possible explanation is the detachment of the products grown on the sample's surface, which leads to the continuous degradation of

Mg. Although DMEM/F-12 supplemented with FBS has many inorganic/organic compounds, and there are many possible matches between the database phases with the experimental data, it is worthy of mentioning that the identification of phases was carried out considering the elements in more significant proportion revealed in the composition analysis by EDS: Mg, O, C.

Moreover, the more complex and secondary compounds identified at more extended times of immersion could be originated from the reaction of the initial degradation products, e.g. $Mg(OH)_2$ and $MgCO_3$, with other compounds of the medium, and the results could be hydrated carbonates and chloride salts. Additionally, in some works, it has been confirmed the presence of calcium and magnesium phosphate by XRD [14], XPS [15], and qualitative elemental mapping obtained by EDS [16]. However, the compositional study carried out by Marco et al. [16] showed that the Ca, P compounds are formed only on the surface and not within the corrosion products layer. The detection of Ca, Na, P by EDS in some zones of the samples agrees with the observation of these elements exclusively on the surface, as these are from DMEM/F-12.

3.3 Mechanical properties

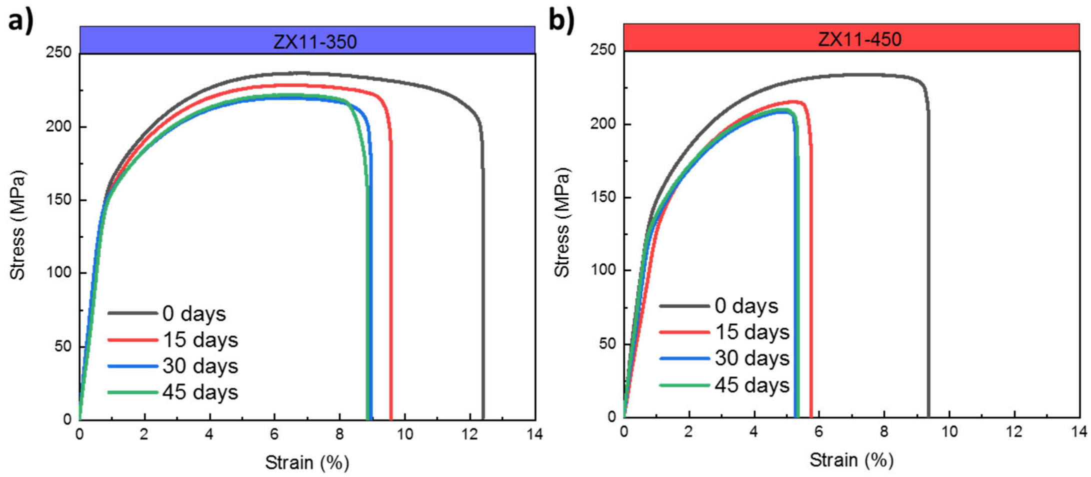

Figure S10. Stress vs strains curves for the a) ZX11-350 and b) ZX11-450 conditions at 0, 15,30 and 45 immersion days in DMEM/F-12 +10% v/v FBS.

3.4 Citotoxicity of the lixiviates

When cells were cultured with diluted alloys lixiviates, and cell viability was evaluated by MTT (Fig. 8a), significantly smaller cell viability, in comparison with the Ctrl, was evidenced for the 5X dilution of the ZX11-350 and ZX11-450 lixiviates at all days of culture, except for the 5X dilution of the ZX11-450 lixiviates at 1 day of cell culture which exhibited a similar viability to that of the Ctrl. This might be explained by the smaller initial degradation rate of ZX11-450 in comparison to ZX11-350, showed by corrosion, Mg ions and H_2 release studies. The decrease observed in cell viability for the 5X dilution of the alloys lixiviates at 3 and 6 days of cell culture, respect to the Ctrl, agrees with the observations from non-diluted lixiviates, where the decrease in cell viability in comparison with Ctrl was even more evident, corroborating that pH rising (as high as 9 for non-diluted extracts at 24 h of alloys immersion in culture medium), and abrupt initial corrosion, with the consequent ions and H_2 release, affect cell viability [17]. As dilution of the lixiviates increased, that is 10X and 15X dilutions, cell viability increased at all culture times showing no significant differences in comparison

with the Ctrl. A trend indicating a higher cell viability for ZX11-450 in comparison to ZX11-350 at all culture times and dilutions was observed; however, differences were not significant, but for dilution 5X at 1 day of culture. On the other hand, when cells were directly observed using the LIVE/DEAD assay, well adhered cells with fusiform morphology and cell to cell contact prolongations were evidenced for all dilutions and culture times. There were no significant differences in the number of viable cells in the Ctrl and that of ZX11-350 and ZX11-350 diluted lixiviates; insert in Fig 8b. Semi-quantitative evaluation of viable cells (insert in Fig 8b), showed that the number of cells increased with culture days for ZX11-350 and ZX11450 diluted lixiviates as for the Ctrl, indicating an adequate proliferation rate for cells in contact with diluted alloys lixiviates.

Similar effects have been observed from previous studies for biocompatible Mg alloys [13], [18]–[20] showing a slight decrease in cell proliferation and metabolic activity [21] upon increasing Mg salts concentration in the medium, with specific effects being dependent on the particular kind of cells tested. Nevertheless, these effects are normally considered as not significant to compromise the biocompatibility of the materials. According to this, and due to the differences for *in vitro* and *in vivo* biological sensitivity to Mg corrosion [22], where it has been demonstrated that corrosion rates *in vivo* can be 1 to 5 times lower than corrosion rates observed *in vitro,* mainly due to the lower concentrations of chloride ions present in blood plasma (103 mM) and bone (48.6–56.7 mM) than in FBS (147.8 mM) [23], we can state that the present alloys, exhibited appropriate cell viability, and showed potential to be used for *in vivo* applications as biodegradable implants in the orthopedic or dental fields.

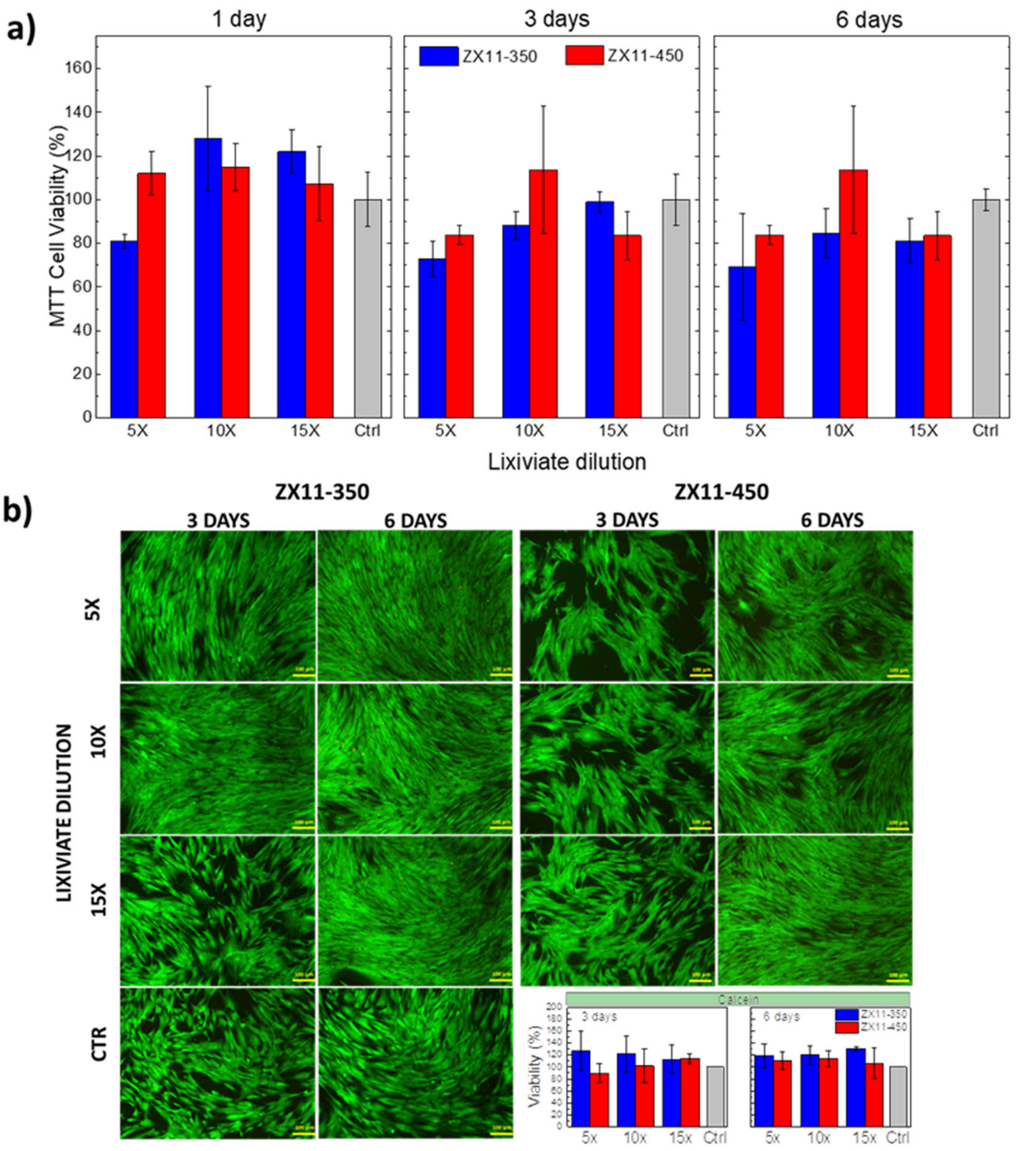

Figure S11. Cell viability in presence of 5X, 10X and 15X diluted alloys lixiviates, as a function of cell culture time, and evaluated from a) MTT and b) LIVE/DEAD assays, where viable and non-viable cells are observed in green and dead respectively. Semiquantitative evaluation of the number of viable alive in comparison to the Ctrl (100% viability) was performed form the micrographs obtained after LIVE/DEAD assays.

3.5 Cititoxicity of the samples without dilution

In order to evaluate the osteoinduction capability of the samples, it was important to evaluate the cytotoxicity of the sampels without dilution. Fig 7a shows that upon culture with non-diluted lixiviates, MTT results exhibited a similar cell viability for ZX11-350, ZX11-450 and Ctrl at 1 day of cell culture. However, by 2 and 6 days of culture, cell viability for ZX11-350 and ZX11-450 was similar, but significantly smaller than that of the Ctrl. This can be mainly ascribed to two different effects. Cell viability, as evaluated from MTT, corresponds to cells metabolic active [24][25], which might then indicate a smaller number of cells and/or a decreased metabolic activity of cells upon culture with ZX11-350 and ZX11-450 lixiviates, but not necessarily a higher dead to alive cells ratio for the alloys lixiviates in comparison to the Ctrl. LIVE/DEAD results shown in Fig 7b, directly exhibits the viable/non-viable cells and their morphology, evidencing a smaller number of cells for ZX11-350 and ZX11-450 lixiviates at 2 and 6 days of cell culture, in comparison with the Ctrl. This can be explained considering that the degradation process of the samples is significant during the first 7 days of immersion, Fig. 3 c) and d), in comparison with longer periods. However, in all cases, 100% of observed

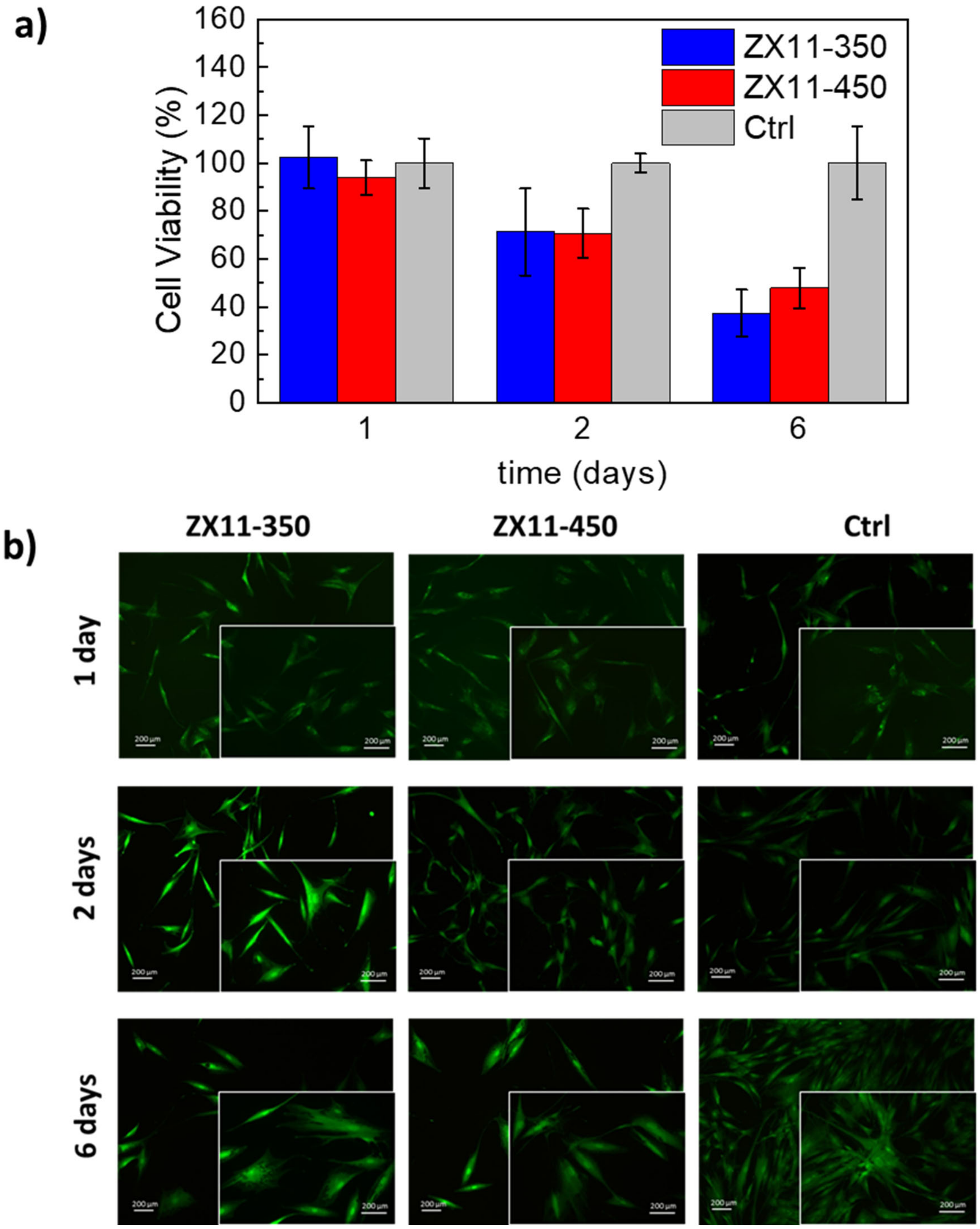

Figure S12. Cell viability in presence of non-diluted alloys lixiviates, as a function of cell culture time, and evaluated from a) MTT and b) LIVE/DEAD assays, where viable and non-viable cells are observed in green and red, respectively.

4 References

[1] B. Millán-Ramos *et al.*, "Biocompatibility and electrochemical evaluation of ZrO2 thin films deposited by reactive magnetron sputtering on MgZnCa alloy," *J. Magnes. Alloy.*, 2021.

[2] D. Mei, S. V. Lamaka, J. Gonzalez, F. Feyerabend, R. Willumeit-Römer, and M. L. Zheludkevich, "The role of individual components of simulated body fluid on the corrosion behavior of commercially pure Mg," *Corros. Sci.*, vol. 147, no. June 2018, pp. 81–93, 2019.

[3] V. Wagener and S. Virtanen, "Protective layer formation on magnesium in cell culture medium," *Mater. Sci. Eng. C*, vol. 63, pp. 341–351, 2016.

[4] D. Mei, S. V. Lamaka, X. Lu, and M. L. Zheludkevich, "Selecting medium for corrosion testing of bioabsorbable magnesium and other metals – A critical review," *Corros. Sci.*, vol. 171, no. April, p. 108722, 2020.

[5] F. Feyerabend *et al.*, "Ion release from magnesium materials in physiological solutions under different oxygen tensions," *J. Mater. Sci. Mater. Med.*, vol. 23, no. 1, pp. 9–24, 2012.

[6] V. Wagener, A. S. Faltz, M. S. Killian, P. Schmuki, and S. Virtanen, "Protein interactions with corroding metal surfaces: Comparison of Mg and Fe," *Faraday Discuss.*, vol. 180, pp. 347–360, 2015.

[7] S. Höhn, S. Virtanen, and A. R. Boccaccini, "Protein adsorption on magnesium and its alloys: A review," *Appl. Surf. Sci.*, vol. 464, no. May 2018, pp. 212–219, 2019.

[8] I. Johnson, W. Jiang, and H. Liu, "The Effects of Serum Proteins on Magnesium Alloy Degradation in Vitro," *Sci. Rep.*, vol. 7, no. 1, pp. 1–14, 2017.

[9] M. A. Khalili and E. Tamjid, "Controlled biodegradation of magnesium alloy in physiological environment by metal organic framework nanocomposite coatings," *Sci. Rep.*, no. 0123456789, pp. 1–13, 2021.

[10] Y. K. Kim *et al.*, "Gas formation and biological effects of biodegradable magnesium in a preclinical and clinical observation," *Sci. Technol. Adv. Mater.*, vol. 19, no. 1, pp. 324–335, 2018.

[11] P. K. Bowen, J. Drelich, and J. Goldman, "Magnesium in the murine artery: Probing the products of corrosion," *Acta Biomater.*, vol. 10, no. 3, pp. 1475–1483, 2014.

[12] H. Ibrahim, A. D. Klarner, B. Poorganji, D. Dean, and A. A. Luo, "Microstructural , mechanical and corrosion characteristics of heat-treated Mg-1 . 2Zn-0 . 5Ca (wt %) alloy for use as resorbable bone fixation material," *J. Mech. Behav. Biomed. Mater.*, vol. 69, no. December 2016, pp. 203–212, 2017.

[13] R. Hou *et al.*, "In vitro evaluation of the ZX11 magnesium alloy as potential bone plate: Degradability and mechanical integrity," *Acta Biomater.*, no. July, 2019.

[14] B. Zhang, Y. Hou, X. Wang, Y. Wang, and L. Geng, “Mechanical properties, degradation performance and cytotoxicity of Mg-Zn-Ca biomedical alloys with different compositions,” *Mater. Sci. Eng. C*, vol. 31, no. 8, pp. 1667–1673, 2011.

[15] D. Tie, F. Feyerabend, N. Hort, R. Willumeit, and D. Hoeche, “XPS studies of magnesium surfaces after exposure to Dulbecco’s modified eagle medium, Hank’s buffered salt solution, and simulated body fluid,” *Adv. Eng. Mater.*, vol. 12, no. 12, pp. 699–704, 2010.

[16] I. Marco *et al.*, “In vivo and in vitro degradation comparison of pure MG, MG-10GD and MG-2AG: A short term study,” *Eur. Cells Mater.*, vol. 33, pp. 90–104, 2017.

[17] A. Mahato *et al.*, “Role of calcium phosphate and bioactive glass coating on in vivo bone healing of new Mg–Zn–Ca implant,” *J. Mater. Sci. Mater. Med.*, vol. 32, no. 5, 2021.

[18] J. Fischer, D. Pröfrock, N. Hort, R. Willumeit, and F. Feyerabend, “Reprint of: Improved cytotoxicity testing of magnesium materials,” *Mater. Sci. Eng. B*, vol. 176, no. 20, pp. 1773–1777, 2011.

[19] R. Amberg, A. Elad, F. Beuer, C. Vogt, J. Bode, and F. Witte, “Effect of physical cues of altered extract media from biodegradable magnesium implants on human gingival fibroblasts,” *Acta Biomater.*, vol. 98, pp. 186–195, 2019.

[20] A. Burmester, B. Luthringer, R. Willumeit, and F. Feyerabend, “Comparison of the reaction of bone-derived cells to enhanced MgCl2-salt concentrations,” *Biomatter*, vol. 4, no. 1, p. e967616, 2014.

[21] T. Zhang *et al.*, “The beneficial influence of microarc oxidation-coated magnesium alloy on the adhesion, proliferation and osteogenic differentiation of bone marrow stromal cells,” *Mater. Lett.*, vol. 137, pp. 362–365, 2014.

[22] H.-S. Han *et al.*, “Current status and outlook on the clinical translation of biodegradable metals,” *Mater. Today*, vol. 23, pp. 57–71, 2019.

[23] A. H. M. Sanchez, B. J. C. Luthringer, F. Feyerabend, and R. Willumeit, “Mg and Mg alloys: How comparable are in vitro and in vivo corrosion rates? A review,” *Acta Biomater.*, vol. 13, pp. 16–31, 2015.

[24] D. Gerlier and N. Thomasset, “Use of MTT colorimetric assay to measure cell activation,” *J. Immunol. Methods*, vol. 94, no. 1, pp. 57–63, 1986.

[25] T. Mosmann, “Rapid colorimetric assay for cellular growth and survival: Application to proliferation and cytotoxicity assays,” *J. Immunol. Methods*, vol. 65, no. 1, pp. 55–63, 1983.
